# The economic burden of Chagas disease: A systematic review

**DOI:** 10.1371/journal.pntd.0011757

**Published:** 2023-11-22

**Authors:** Mônica Viegas Andrade, Kenya Valéria Micaela de Souza Noronha, Aline de Souza, André Soares Motta-Santos, Paulo Estevão Franco Braga, Henrique Bracarense, Maria Carolina Corrêa de Miranda, Bruno Ramos Nascimento, Israel Molina, Francisco Rogerlândio Martins-Melo, Pablo Perel, Yvonne Geissbühler, Monica Quijano, Isis Eloah Machado, Antônio Luiz Pinho Ribeiro

**Affiliations:** 1 Department of Economics, Universidade Federal de Minas Gerais, Belo Horizonte, Brazil; 2 Faculty of Economic Sciences, Universidade Federal de Minas Gerais, Belo Horizonte, Brazil; 3 Center for Health Technology Assessment, Universidade Federal de Minas Gerais, Belo Horizonte, Brazil; 4 Center for Development and Regional Planning, Universidade Federal de Minas Gerais, Belo Horizonte, Brazil; 5 Department of Internal Medicine, Faculty of Medicine, Universidade Federal de Minas Gerais, Belo Horizonte, Brazil; 6 Telehealth Center and Cardiology Service, Hospital das Clínicas, Universidade Federal de Minas Gerais, Belo Horizonte, Brazil; 7 International Health Unit Vall d’Hebron-Drassanes, Infectious Diseases Department, Vall d’Hebron University Hospital, Barcelona, Spain; 8 Federal Institute of Education, Science and Technology of Ceará, Fortaleza, Brazil; 9 World Heart Federation, Geneva, Switzerland; 10 Novartis Global Health, Basel, Switzerland; 11 Department of Family Medicine, Mental and Collective Health, Universidade Federal de Ouro Preto, Ouro Preto, Brazil; Inkosi Albert Luthuli Central Hospital, SOUTH AFRICA

## Abstract

**Background:**

Chagas disease (CD) is a neglected disease affecting millions worldwide, yet little is known about its economic burden. This systematic review is part of RAISE project, a broader study that aims to estimate the global prevalence, mortality, and health and economic burden attributable to chronic CD and Chronic Chagas cardiomyopathy. The objective of this study was to assess the main costs associated with the treatment of CD in both endemic and non-endemic countries.

**Methods:**

An electronic search of the Medline, Lilacs, and Embase databases was conducted until 31st, 2022, to identify and select economic studies that evaluated treatment costs of CD. No restrictions on place or language were made. Complete or partial economic analyses were included.

**Results:**

Fifteen studies were included, with two-thirds referring to endemic countries. The most commonly investigated cost components were inpatient care, exams, surgeries, consultation, drugs, and pacemakers. However, significant heterogeneity in the estimation methods and presentation of data was observed, highlighting the absence of standardization in the measurement methods and cost components. The most common component analyzed using the same metric was hospitalization. The mean annual hospital cost per patient ranges from $25.47 purchasing power parity US dollars (PPP-USD) to $18,823.74 PPP-USD, and the median value was $324.44 PPP-USD. The lifetime hospital cost per patient varies from $209,44 PPP-USD for general care to $14,351.68 PPP-USD for patients with heart failure.

**Discussion:**

Despite the limitations of the included studies, this study is the first systematic review of the costs of CD treatment. The findings underscore the importance of standardizing the measurement methods and cost components for estimating the economic burden of CD and improving the comparability of cost components magnitude and cost composition analysis. Finally, assessing the economic burden is essential for public policies designed to eliminate CD, given the continued neglect of this disease.

## Author summary

Chagas disease is a global health concern with significant economic implications, yet our understanding of its economic burden remains limited. As part of the RAISE project, aimed at estimating the global impact of chronic Chagas disease and chronic Chagas cardiomyopathy, we conducted a systematic review to assess the costs associated with Chagas Disease treatment in both endemic and non-endemic countries. We identified fifteen studies. The most commonly investigated cost components included inpatient care, exams, surgeries, consultations, drugs, and pacemakers. We found substantial heterogeneity in estimation methods and data presentation, revealing a lack of measurement and cost components standardization. Our systematic review represents the first comprehensive assessment of Chagas Disease treatment costs. Our findings emphasize the need for standardized measurement methods and cost components to improve comparability and facilitate economic burden estimation. The high costs of invasive treatments for cardiomyopathies remain challenging for low and middle-income countries. Estimating the economic burden of Chagas disease provides crucial information for conducting policy evaluations and interventions. For instance, these studies can support international agencies in funding research for vaccine development and improved vector control programs. While vaccine research is in its early stages, promising antiparasitic therapies offer potential for disease control.

## 1 Introduction

Chagas disease (American trypanosomiasis) is a life-threatening illness caused by the protozoan *Trypanosoma cruzi* and transmitted to humans mainly by contact with feces/urine of infected blood-sucking triatomine bugs (vector-borne transmission). The disease can also be transmitted through blood transfusion, organ transplant, contaminated food or drink (oral transmission), mother-to-child transmission during pregnancy or breastfeeding, or laboratory accidents [1].

Chagas disease encompasses two distinct clinical phases: acute and chronic. The acute phase is characterized by mild and nonspecific symptoms, such as fever, headaches, enlarged lymph glands, pallor, and muscle pain. Severe manifestations in this phase such as myocarditis, pericardial effusion, and meningoencephalitis are infrequent and affect only 1 to 5% of patients. In the chronic phase, most patients (approximately 60–70% of cases) remain asymptomatic, known as the indeterminate form. However, within a period of 10 to 30 years after infection, 30–40% of patients in the chronic phase can develop visceral complications which include cardiomyopathy and megaviscera. Additionally, mild polyneuropathy may be present in 10% of cases [2].

Chagas disease is endemic in 21 Latin American countries, where it remains an important public health problem and one of the most significant neglected tropical diseases (NTDs) [3,4]. Despite this importance, uncertainty concerning mortality, prevalence, and sequelae data is still an issue mainly due to the underreporting and misclassification [5]. It is estimated that about 6–7 million people are infected with *T*. *cruzi* worldwide, mostly in Latin America, and an estimated 75 million people are at risk of infection [6]. Globally, Chagas disease causes approximately 9,500–12,000 deaths annually [3]. Brazil is the country with the largest estimated number of cases, around 2 million cases representing about 1% of the population, and the highest mortality [3]. With the intensification of international migration flows, the disease has spread to non-endemic areas of the world, increasing the interest of the international community [3,7,8]. For instance, the Center for Disease Control and Prevention (CDC) estimates that 300,000 people live with Chagas Disease in the USA [9].

The treatment of Chagas disease is based on two main approaches: aetiological treatment and management of medical complications. Aetiological treatment is done with antiparasitic drugs such as benznidazole or nifurtimox. While they are very effective for acute and recent infection, and for the prevention of maternofoetal transmission, their efficacy declines in people who have chronic infection, especially those older than 18 years of age [10]. In the presence of visceral involvement, parasiticidal treatment is of little or no value. The safety profile of both drugs is far from ideal, with frequent adverse events and high rates of drug discontinuation, mainly in adults [9–13]. Both drugs are very effective if given in the beginning of the acute phase, but the effectiveness falls the longer the patient stays without treatment [11,14].

For individuals who have progressed to the chronic form of the disease, management is focused on treating symptoms and preventing further damage to the heart and digestive system [9]. Chagas disease has a costly management, specially later in life [15–17]. Patients with chronic Chagas cardiomyopathy (CCM) might require transplants, pacemaker implants, surgeries, and hospitalization [11,18].

Besides medical direct costs, Chagas disease has been associated with high indirect costs related to early mortality, disability, and loss of work capacity [3,19]. The global burden of disease is over 275,000 disability-adjusted life years (DALYs) lost in 2019 [20]. In Latin America, 752,000 working days are lost due to Chagas disease-associated premature deaths [4]. Studies that evaluate the costs associated with disease control and treatment are key to supporting public policies that enable the planning of disease management and elimination strategies. A systematic evaluation of the costs of managing the disease is lacking in the medical literature. In this study, we conducted a systematic review to assess the main costs of the treatment of Chagas disease in endemic and non-endemic areas. This study is part of a broader project (The bu**R**den of Ch**A**gas d**ISE**ase in the contemporary world: the RAISE project) that aims to estimate the global and country-specific prevalence, mortality, and health and economic burden attributable to chronic CD and Chronic Chagas cardiomyopathy.

## 2 Methods

### 2.1 Study design

This systematic review (SR) was conducted to summarize the studies assessing the costs associated with treatment of Chagas disease worldwide. This report followed the principles of the Preferred Reporting Items for Systematic reviews and Meta-Analyses (PRISMA) statement (**S1 Table**) [21] and was registered in PROSPERO (CRD42022335116).

### 2.2 Research question

The SR aims to answer the question: how much are Chagas disease’s direct and indirect costs? Direct costs encompass all expenses associated with treatment, including both medical and non-medical costs, regardless of the stakeholder involved. Typical medical costs comprise hospitalizations, diagnostic tests, medications, consultations, surgical procedures, as well as other necessary materials and inputs. Non-medical costs usually include expenses for food, accommodation, and transportation incurred while seeking or receiving diagnosis and treatment. Indirect costs are the productivity loss and opportunity costs resulting from the disease.

**Fig 1:**
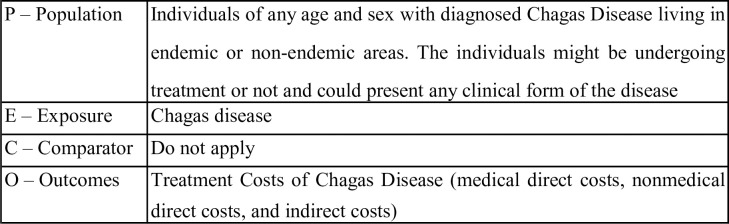
PECO research question.

The research question in PECO format is described below in **Fig 1**.

### 2.3 Search strategy and eligibility criteria

Structured electronic searches of the Medline (via PubMed), Lilacs (via BVS), and Embase databases were conducted on October 5^th^, 2022, to identify and select economic studies that evaluated treatment costs of Chagas disease. The search included references published until May 31^st^, 2022. The descriptors “Costs and Cost Analysis”, “Economics”, “Cost Allocation”, “Health Care Costs”, “Chagas Disease”, “American Trypanosomiasis”, and several synonyms and alternative terms were used. The full search strategies are available in **S2 Table**. A complementary search in scientific journals, thesis and dissertation databases, and conference abstracts was performed to identify possible important references that have not been included. The references were exported to EndNote X7.5 and transferred to the Rayyan QCRI online application [22] for the selection process.

Complete or partial economic analyses that evaluated the costs of Chagas disease were included. Different perspectives were considered (healthcare system, household, or societal). No restrictions on the initial date, place, or language were made. Letters, brief commentaries, papers without a published full text, studies about control and prevention programs, and cost analyses of Chagas disease combined with others infectious diseases were excluded. Cost-effectiveness studies were included only when the costs of illness were estimated by the authors themselves.

### 2.4 Study selection and data collection

The selection of studies was conducted in two phases. Phase I was a screening of the titles and abstracts. After phase I, the records were retrieved, and the full texts were assessed in phase II. Phase III was data collection.

A spreadsheet was designed *a priori* for data collection (**S3 Table**). To assemble this spreadsheet, some reasonably comprehensive articles were assessed. This assessment allowed the authors to identify the cost items to be collected. These items formed a standardized table of cost components and their characteristics (e.g., metric, periodicity, phase of the disease, and unit of measurement). The spreadsheet included information on the country of origin, type of study (cost of illness, economic evaluation, epidemiological), study perspective (societal, healthcare system, both healthcare system and household, institution, only indirect costs), currency-year, costs, stages of the disease (acute or chronic), form of the disease (indeterminate, cardiac, digestive, or mixed), and sponsor (none, national, international, or both). The societal perspective includes both direct and indirect costs, while the healthcare system and household perspectives consider only direct costs. The institutional perspective refers to studies that evaluate the costs of specific health center units. Other relevant information was collected using the open form. All phases were conducted by at least two independent researchers and discrepancies were resolved by consensus [23].

### 2.5 Data analysis

#### 2.5.1 Qualitative analysis

Initially, the studies were characterized according to the year of publication, period of analysis, country, target population, study perspective, type of study, method of cost estimation (model-based/non-model-based), phase and form of the Chagas disease, and funding source. Maps were built to show the geographical location of the articles. The qualitative analysis focused on demonstrating a pattern in terms of the components of cost evaluated in the studies. The cost items were reclassified into the usual three major cost categories: (i) medical direct costs; (ii) nonmedical direct costs (food, lodging, and travel costs); and (iii) indirect costs (absenteeism, presenteeism, and mortality). The medical direct costs comprise the following components: Screening, Diagnosis, Consultation, Ambulatory/Outpatient care, Benznidazole/Nifurtimox, Amiodarone, Digoxin, Drugs/Medicines (Not specified), Exams, Material/Inputs, Emergency, Hospitalization, Surgery (Digestive), Surgery (Not specified), Pacemaker, Defibrillator, and Transplant.

#### 2.5.2 Quantitative analysis

Median, minimum, and maximum values were used to describe the costs of selected components (hospitalization, pacemaker, surgery, and transplant). Studies that only reported values for the total population were excluded from the quantitative analysis as these figures are conditional on the population size. For this analysis, the monetary values as presented in each study were converted into 2022 purchasing power parity US dollars (PPP-USD) using the purchase power parity (PPP) conversion rate calculated by the International Monetary Fund (IMF). The conversion was performed in the online The Campbell and Cochrane Economics Methods Group and the Evidence for Policy and Practice Information (CCEMG-EPPI-Center Cost Converter) tool [24]. Data collection and tables were constructed in Microsoft Excel. R [25] and Stata 14 were used to summarize the data.

### 2.6 Quality assessment

There is no widely accepted instrument to evaluate the quality of economic studies. Nevertheless, an instrument to estimate the quality of the reports was developed from other checklists [26–28], including a specific question about disaggregation of data according to Chagas disease´s phases and forms (**S4 Table**). Two independent researchers conducted this assessment, and the discrepancies were solved by consensus.

The quality assessment was divided into three components: analytical framework, methodology and data, and analysis and reporting. The analytical framework pertains to overarching aspects of the study, such as the suitability of the research question and the chosen target population. The methodology and data refer to the description of the methods employed in conducting the study, including questions about the cost components used and the approach for their calculation. The analysis and report pertain to the outcomes presented in the articles, such as the detailed breakdown of costs.

## 3 Results

### 3.1 Study selection

**Fig 2:**
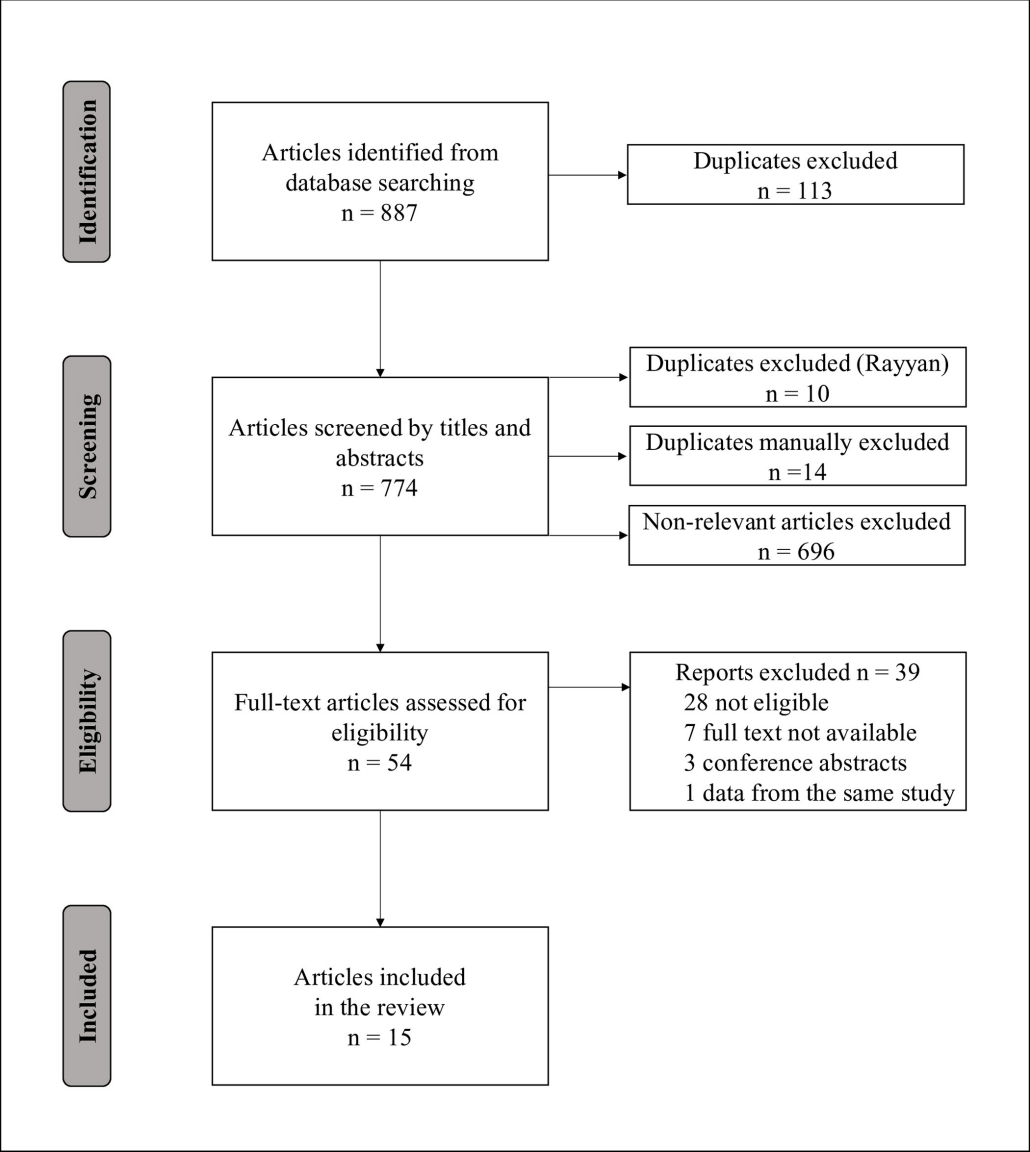
Flow diagram of the systematic review selection.

The electronic search returned 774 records after duplicate removal. Of these, 720 were excluded in phase I. Most studies were excluded by publication type, scope, and outcome data availability. Therefore, 54 references were selected for complete review. Of these, 15 were included in the final analysis, 28 were considered not eligible, three were conference abstracts, and seven were unavailable. We contacted the authors to request the full versions. Only two authors answered the request, but the papers were not available. Two references were based on the same study; therefore, the most complete paper was included in the analysis [18,29] (**Fig 2**). The reasons for exclusion in phase II were: 1) studies that cost estimations were only based on other published papers (N = 17); 2) interventional studies which cost estimates were specific to programs, drugs, or technologies (N = 7); 3) commentary/letters (N = 2); and 4) papers without cost estimations (N = 2). The excluded and included studies list can be found in **S5 and S6 Tables.**

### 3.2 Characteristics of the included studies

**Fig 3:**
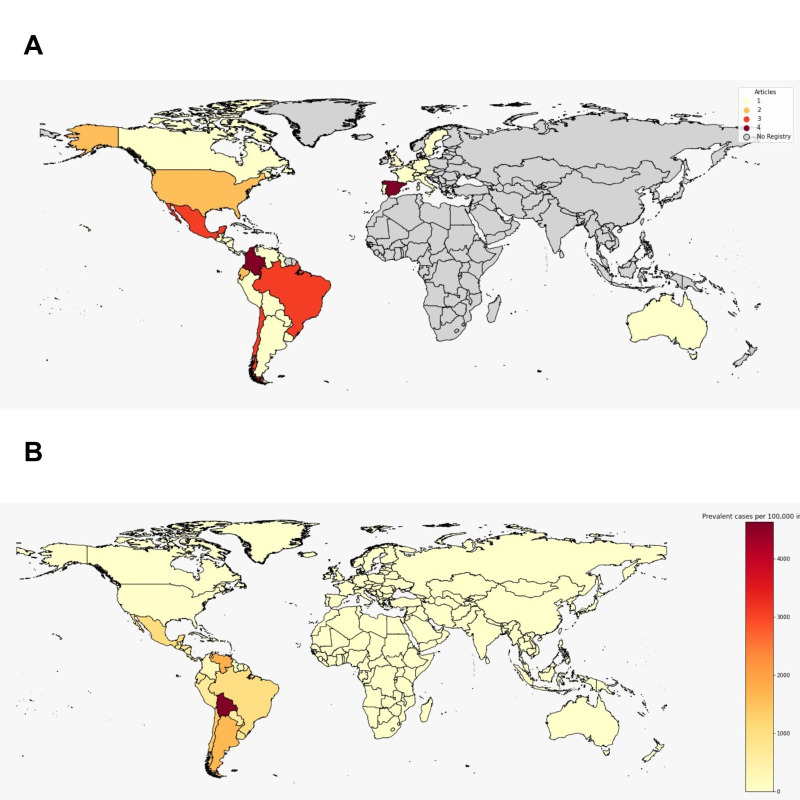
Distribution of articles included in the Systematic Review by country of analysis **(A)** and estimated prevalence of Chagas Disease per 100,000 inhabitants in 2019 **(B).** Note: Maps were created using Geopandas package within Python environment, version 3.10.0. The base map used is derived from an openly available ESRI shape file source (https://hub.arcgis.com/datasets/esri::world-countries-generalized). Prevalence data are available at IHME (https://vizhub.healthdata.org/gbd-compare/), and investigated countries were collected from the 15 studies included in the systematic review.

The selected studies were published between 1998 and 2021, with the majority concentrated between 2011 and 2020. The included papers started their data collection between 1992 and 2017. Four studies did not report the period of analysis. Seven countries were represented: Brazil (N = 3), Chile (N = 3), Colombia (N = 4), Ecuador (N = 2), Mexico (N = 3), Spain (N = 4), and the USA (N = 2) (**Fig 3A**). Two-thirds of the studies refer to endemic countries for Chagas [30]. Only one study estimated the global economic burden of Chagas disease, considering the 33 countries with notified cases in the last 15 years [31] **(Fig 3A and 3B)**.

Most studies were cost of illness (N = 6), followed by economic evaluations (N = 5), epidemiological (N = 2), and mixed studies (epidemiological and cost of illness) (N = 2). Six studies estimated the costs considering the general population, three specifically to mothers and children/newborns, and six to hospital patients diagnosed with *Trypanosoma cruzi*. Among the six papers focusing on the population receiving inpatient care, the number of patients ranged from 13 in Mexico to 1729 based on hospitalization records in Spain. The most common perspectives were societal (N = 6) and healthcare system (N = 5) followed by the perspectives of the institution (N = 3). One study only analyzed the indirect costs related to mortality. Six studies were model-based: three were Markov models, two were decision tree models, and one was a micro-costing study. Six full texts reported funding sources, four of them only by national funds, one by an international fund, and one presented both types of funding. International resources were only reported in two studies. Ten studies disaggregated the cost by the phases of the disease. Among them five only calculated the costs considering the chronic phase and five also included the acute phase **(Table 1)**.

**Table 1:** Attributes of the studies included in the systematic review (n = 15).

Decade of publication	n	%	Reference
1991–2000	1	6.67	[32]
2001–2010	3	20.00	[18,33,34]
2011–2020	10	66.67	[7,19,31,35–41]
2021	1	6.67	[17]
Decade of the analysis start			
1991–2000	3	20.00	[7,32,34]
2001–2010	5	33.33	[17,18,19,35,36]
2011–2020	3	20.00	[37–39]
NA/NI	4	26.67	[31,33,40,41]
Type of study			
Cost of illness	6	40.00	[18,31,34,35,39,40]
Epidemiological study	2	13.33	[7,36]
Economic evaluation	5	33.33	[17, 19,37,38,41]
Cost-effectiveness	1	20.00	[41]
Cost-utility	1	20.00	[38]
Cost-effectiveness and Cost-utility	1	20.00	[37]
Microcosting	1	20.00	[19]
Only indirect costs	1	20.00	[17]
Both (Epidemiological and cost of illness)	2	13.33	[32,33]
Target population			
General population	6	40.00	[17,31–33,39]
Mothers and Newborns/children	3	20.00	[37,38,41]
Hospital patients	6	40.00	[7,18,19,34–36]
Study perspective			
Health Care System (only)	5	33.33	[7,18,32,36]
Institution (only)	3	20.00	[19,34,35]
Societal (direct and indirect costs)	6	40.00	[31,33,38–41]
Indirect costs (only)	1	6.67	[17]
Estimation of resources and costs			
Model-based	6	40.00	[19,31,37,38,40,41]
Non-Model-based	9	60.00	[7,17,18,32–36,39]
Funding source			
No	9	60.00	[7,17,19,32,34–36,38,41]
Yes	6	40.00	[18,31,33,37,39,40]
Nacional (governmental)	4	66.67	[33,37,39,40]
Internacional (non-profit organization)	1	16.67	[18]
Both	1	16.67	[31]
Separation of the disease by phases/forms			
No	5	33.33	[7,17,19,36,39]
Yes	10	66.67	[18,31–35,37,38,40,41]
Acute and Chronic	5	50.00	[31,33,37,38,40]
Acute, Cardiac, Digestive, and Indeterminate	4	80.00	[31,33,38,40]
Acute, Cardiac, Digestive, Indeterminate, and Mixed	1	20.00	[37]
Chronic (only)	5	50.00	[18,32,34,35,41]
Cardiac	4	80.00	[18,32,34,35]
Cardiac, Digestive, and Indeterminate	1	20.00	[41]

### 3.3 Quality assessment

Most reports adequately stated their objectives (N = 14/15, 93.3%), described the target population (N = 13/15, 86.7%), and stated the perspective adopted (N = 14/15, 93.3%). The time horizon was not assessed in six studies as they performed transversal analysis. Seven of nine studies reported adequate time horizons (N = 7/9, 77.8%) and one was classified as inadequate and unclear in another. 86.7% of the studies clearly described the methods for cost estimation (N = 13/15). In two studies, this criterion was only considered partially fulfilled. Almost all analyses included cost components in line with the study perspective (N = 14, 93.3%). In 40% of the studies, the cost components were considered to be clearly described (N = 6/15). In the others, this criterion was only partially fulfilled (N = 7/15, 46.7%) or not at all (N = 2/15, 13.3%). Information on the currency and the period in which the costs were collected were available in 86.7% of the reports (N = 13). Adjustment for inflation was not applicable in six studies. For the remaining nine studies only three made this adjustment (33.3%); it was not done in five, and it was unclear in one. About half the studies included a discount rate (N = 8/15, 53.3%). Only about half the studies included productivity costs (N = 7/15, 46.7%). 40% of the studies divided the cost analysis considering different disease phases (N = 6/15) and 26.6% (N = 4) focused on chronic cardiac form. Only 33.3% presented the cost results disaggregated by components (N = 5/15). A sensitivity analysis was conducted in 40% of the studies (N = 6/15). The generalization of the results was only discussed in 26.7% of the reports (N = 4/15). Conflicts of interest were stated in most reports (N = 9/15, 60%). The results of the quality assessment are available in **Table 2.** The analysis disaggregated by study is available in the **S7 Table**.

**Table 2:** Quality assessment of the included studies.

Items Assessed	Number of studies (%)				
	Yes	No	Partially	Unclear	Not applicable
Objective clearly stated and properly answered	14 (93.3%)	−	1 (6.7%)	−	−
Target population clearly described	13 (86.7%)	−	2 (13.3%)	−	−
Study perspective stated	14 (93.3%)	1 (6.7%)	−	−	−
Time horizon appropriate	7 (46.7%)	1 (6.7%)	−	1 (6.7%)	6 (40.0%)
Method for costs estimation clearly described	13 (86.7%)	−	2 (13.3%)	−	−
Cost components in line with the study perspective	14 (93.3%)	−	1 (6.7%)	−	−
Cost components clearly described	6 (40.0%)	2 (13.3%)	7 (46.7%)	−	−
Information on the currency and the period in which the costs were collected	13 (86.7%)	2 (13.3%)	−	−	−
Adjustment for inflation	3 (20.0%)	5 (33.3%)	−	1 (6.7%)	6 (40.0%)
Discount rate	8 (53.3%)	5 (33.3%)	−	−	2 (13.3%)
Productivity costs stated	7 (46.7%)	8 (53.3%)	−	−	−
Separation in disease phases/forms	6 (40.0%)	5 (33.3%)	4 (26.7%)	−	−
Cost components results presented in a disaggregated way	5 (33.3%)	3 (20.0%)	4 (26.7%)	−	3 (20.0%)
Sensitivity analysis performed	6 (40.0%)	9 (60.0%)	−	−	−
Generalization of the results discussed	4 (26.7%)	10 (66.7%)	1 (6.7%)	−	−
Conflict of interest stated	9 (60.0%)	6 (40.0%)	−	−	−

### 3.4 Qualitative synthesis: cost components

Chagas disease has a complex line of care, including inpatient and outpatient care, various diagnosis and follow-up exams, and high-complexity medical procedures. It is not easy to provide a comprehensive cost estimate in a single study. Studies commonly focus on certain aspects of the treatment, especially the cardiac, cardiomyopathic, and/or digestive forms. In this review, direct medical costs were the most evaluated (N = 13) in the included studies, while direct non-medical costs (N = 2) were rarer. The direct medical cost components were classified into 17 categories. Among them, the most investigated were inpatient care (N = 11), exams (N = 9), surgeries (N = 7), consultation (N = 6), drugs (N = 6), and pacemakers (N = 5). Transplants were only present in two analyses. Screening and diagnosis costs were considered in five studies. The total direct medical costs were reported by seven analyses. Among the studies that analyzed non-medical costs, one did a more comprehensive analysis, including food, accommodation, and travel costs, while the other included only travel costs. The indirect costs were present in seven studies. The total cost estimate (direct+indirect) was available in five references (**Table 3**).

**Table 3:** Cost components included in the selected articles.

Cost component	Study															
	7	17	18	19	31	32	33	34	35	36	37	38	39	40	41	Total
Medical direct cost																
Screening										✓			✓			2
Diagnosis		✓			✓					✓		✓				4
Consultation			✓							✓	✓	✓	✓	✓		6
Ambulatory/Outpatient care		✓	✓													2
Benznidazole/Nifurtimox										✓						1
Amiodarone					✓					✓				✓		3
Digoxin					✓						✓					2
Drugs/Medicines (Not specified)		✓			✓			✓			✓		✓	✓		6
Exams		✓	✓		✓			✓		✓	✓	✓	✓	✓		9
Material/Inputs					✓											1
Emergency		✓										✓				2
Hospitalization	✓	✓	✓		✓		✓	✓	✓	✓	✓	✓	✓			11
Surgery (Digestive)					✓					✓	✓		✓	✓		5
Surgery (Not specified)		✓						✓								2
Pacemaker					✓		✓			✓	✓			✓		5
Defibrillator											✓					1
Transplant											✓			✓		2
Total (Medical direct cost)		✓		✓	✓		✓			✓	✓	✓				7
Non-medical direct cost																
Food												✓				1
Lodging												✓				1
Traveling costs (Bus fare)											✓	✓				2
Total (Non-medical direct cost)											✓	✓				2
Indirect cost																
Indirect costs				✓		✓					✓	✓	✓	✓	✓	7
Total cost																
Total (Direct and indirect cost)				✓		✓						✓	✓	✓		5

### 3.5 Quantitative analysis: Costs estimation

Much heterogeneity in the estimation methods and presentation of data was observed. The heterogeneity in cost estimations was evaluated considering the following attributes: statistics (mean, median, minimum and maximum value, and total); unity of analysis (episode, patient, unity, and total), periodicity (annual, lifetime, and per period); phase of the disease (acute and chronic); and the form of chronic disease (cardiac, cardiomyopathy, digestive, heart failure, mega-colon, mega-esophagus, mixed, and indeterminate). To evaluate the cost metrics, the information on the statistics, unity of analysis, and periodicity reported by the articles were combined. **S8 Table** presents the number of papers that estimated at least one cost component by each type of metric. The most common metric is the mean annual cost per patient (N = 8), followed by the total annual cost for the population (N = 5).

To ensure comparability among the cost estimate papers, they were categorized based on the metrics they reported. The selection process involved choosing papers that utilized the most used metrics for each component (**Table 4**). Furthermore, this analysis focused exclusively on studies that provided cost information for the chronic phase. The components examined in this analysis included hospitalization, surgery, pacemaker, and transplant. Even for the most prevalent metrics, the number of papers that analyzed each component is small. For instance, for the most common component analyzed, hospitalization, the number of studies using the same metric is only four. Four articles reported the mean annual hospital cost per patient and provided 18 different estimates. Each estimate refers to a specific context (country/region, bed type, and chronic disease form). The annual hospitalization cost ranges from $25.47 PPP-USD [40] to $18,823.74 PPP-USD [34], and the median value was $324.44 PPP-USD. The two extreme values refer to studies conducted in Mexico but in different contexts. The minimum value regards general hospitalization, while the maximum refers to a cardiac/cardiopathy form of patients who received emergency care and used high-cost inputs. Excluding this study, the maximum value reduces to $710.92 PPP-USD in Colombia [18] for patients with heart failure receiving ICU care. The lifetime hospital cost per patient was estimated in only two studies [38,40] for Mexico and Spain. The magnitude varies from $209,44 PPP-USD [40] for general care to $14,351.68 PPP-USD [38] for patients with heart failure.

**Table 4:** Summary statistics of selected cost components for the chronic phase of Chagas disease (2022 US$ PPP).

Metric	Median	Country	Min	Country	Max	Country	SD	# of estimations	ID
Hospitalization									
Annual mean per patient	324.44	Colombia	25.47	Mexico	18,823.74	Mexico	6,108.75	18	[18,32,4,40]
Lifetime mean per patient	832.41	Mexico	209.44	Mexico	14,351.68	Spain	5,186.66	10	[38,40]
Annual total per patient	274.18	Chile	11.12	Chile	1,085.29	Chile	355.36	8	[37]
Surgery									
Annual mean per patient	3,722.47	Colombia	2,845.80	Colombia	4,599.14	Colombia	1,239.80	2	[18]
Lifetime total per patient	16,756.21	USA	5,229.64	USA	50,531.34	USA	20,069.22	6	[41]
Pacemaker									
Lifetime total per patient	25,119.90	USA	10,348.54	USA	25,119.90	USA	8,528.25	3	[41]
Transplant									
Lifetime total per patient	1,065,398.00	USA	438,929.30	USA	1,065,398.00	USA	361,691.80	3	[41]
Indirect costs									
Lifetime mean per patient	1,422.44	Sapin	185.76	Spain	9,027.34	Mexico	2,808.23	11	[38,40]
Lifetime total per patient	205,504.60	USA	36,045.87	USA	1,066,757.00	USA	334,474.50	8	[41]

The number of studies reporting the same metric for surgery, pacemakers, and transplants is even smaller. No more than one article uses the same metric, making the comparison unfeasible. Castillo-Riquelme et al [18]. estimated the mean annual surgery cost per patient for Colombia considering two forms of chronic Chagas disease: heart failure ($4,599.14 PPP-USD) and cardiomyopathy ($2,845.80 PPP-USD). Stillwaggon et al [41]. estimated the lifetime costs of Chagas disease in the United States, including the pacemaker and transplants. According to their results, the lifetime pacemaker cost per patient varies from $10,348.54 PPP-USD (for babies/newborns) to $25,119.90 PPP-USD (for mothers). These values were much higher for transplants, $438,929.30 PPP-USD (for babies/newborns) and $1,065,398.00 PPP-USD (for mothers).

For indirect costs, the most prevalent metric was the lifetime average cost per patient (11 estimates in two studies), followed by the lifetime total per patient (eight estimations in one study). The lifetime average cost per patient exhibited great variation, from 185.76 PPP-USD to 9,027.34 PPP-USD due to absenteeism in Spain and Mexico, respectively. The lower value refers to lost productivity due to medical visits received by patients with chronic digestive Chagas disease throughout their lives [38]. The upper bound estimated [40] is the lifetime average indirect cost per chronic patient due to workdays lost. The lifetime total cost per patient due to premature mortality varied from 36,045.87 PPP-USD to 1,066,757.00 PPP-USD in the United States [41]. The first value regards the productivity loss of undiagnosed babies while the higher value refers to very severe chronic cardiac disease for diagnosed mothers.

The only study that allowed a more comprehensive comparison among the countries was conducted by Lee et al [31]. It includes 33 countries with Chagas cases in the last 15 years. The authors built a Markov model with 1-year cycles and five health states (acute, indeterminate, cardiomyopathy with or without congestive heart failure, mega-viscera, and death). Based on empirical evidence, the authors conducted estimations of the annual cost per patient for each stage of Chagas disease. Among the three income groups, congestive heart failure was identified as the most expensive stage, followed by mega viscera and cardiomyopathy. Notably, the expenditure associated with congestive heart failure was six times higher than the acute expenditures for low-income countries, and nearly twice as high for high-income countries. The model was run for four groups of countries classified according to their per capita income: low income (GDP per capita<US$5,053), low-middle income (US$5,053-US$11,171), high-middle income (US$11,171-US$39,615) and high income (>$39,615). The economic burden per case for each group of countries was estimated by multiplying the average cost by the number of cases, considering indirect costs (absenteeism, premature death, and presenteeism) and direct medical costs. DALYs were converted into lost productivity. The main results showed that the annual health costs of an infected individual are $564.06 PPP-USD and 0.51 DALYs. Globally, the annual burden of disease was estimated at $746.7 million PPP-ISD and 806,170 DALYs. The annual healthcare by patients with chronic Chagas disease varies from $455.77 PPP-USD in Latin American countries to $2,572.78 PPP-USD in USA, Canada, and Australia. The lifetime costs per patient vary from $3,094 PPP-USD to $23,221.66 PPP-USD, respectively.

## 4 Discussion

To the best of our knowledge, this is the first SR of the literature on the costs of Chagas disease treatment. The complexity of the disease reflects on the broad spectrum of procedures and healthcare services required to manage the disease. Most studies focus on the chronic phase, specifically cardiac, cardiomyopathic, and digestive forms, as it includes the most expensive procedures. Medical direct costs were more frequently investigated among the cost components, especially inpatient care, exams, surgeries, consultations, drugs, and pacemakers. The systematic review revealed significant heterogeneity and variance in reported costs across studies, primarily due to the absence of standardization in the measurement methods and cost components included. The heterogeneity in cost estimations encompasses the statistics used in the results presentation, the unity of analysis, periodicity, phase of the disease, and the form of chronic disease. This heterogeneity presents a challenge in comparing costs across studies and drawing conclusions that can be generalized to other settings. Additionally, comparing cost compositions among different health systems and economies was not possible. Conducting cost composition analyses would facilitate comparisons among different care protocols. This heterogeneity has been noted in other SR of cost-of-illness studies [26,42,43]. For instance, Lesyuk et al [42]. conducted a SR of cost-of-illness studies related to heart failure and found significant variability in the cost components considered and the estimates obtained. The total annual costs per patient ranged from US$868 (2016 PPP) in South Korea to US$25,532 (2016 PPP) in Germany. Similarly, Ribeiro-Rotta et al [43]. conducted a SR of cost-of-illness studies related to oral cancer and found considerable variability among studies, particularly regarding the surveyed cancer sites, the cost estimation methods, and the cost components included. According to the authors, the comparison among the cost estimates was not possible. For infectious diseases, Andrade et al [26]. conducted a SR of the economic burden of malaria. Even for acute malaria episodes that require fewer complex procedures, there was no standardization in the methods used to estimate costs, and the cost components varied significantly. The magnitude of costs differed primarily due to differences in the organization of healthcare systems.

Some studies only focus on specific institutions, which limits their external validation. Abuhab et al [35]. analyzed the costs of the first hospitalization of patients with Chagas disease who were followed for five years in a high-complexity cardiology university hospital in São Paulo, Brazil. The authors compared the average value of the first hospitalization between patients with Chagas cardiomyopathy and non-Chagas patients with heart failure. Hasslocher-Moreno et al [19]. used a micro-costing approach to estimate the costs of Chagas disease care at the Laboratory of Clinical Research in Chagas Disease of Fiocruz Foundation in Brazil. The investigation covered all the activities and procedures involved in providing comprehensive patient care. Vallejo et al [34]. conducted a retrospective study to estimate the costs associated with chronic Chagas heart disease in a cardiology hospital in Mexico. The study analyzed the medical records of 13 patients and the costs were valued according to the hospital’s cost system.

The target population of the studies is either Latin American residents in their country or immigrants in non-endemic countries. Specifically, some studies were conducted in the United States (N = 1) [41] and Spain (N = 3) [7,36,38], which receive the largest contingent of immigrants from Latin America [36]. A significant proportion of the studies target specific populations (7 of 15). Among them, three analyzed mothers and newborns since congenital transmission of CD is the primary form of disease dissemination in non-endemic countries [11,36]. These papers estimated the costs of CD to evaluate screening protocols [37,38,41]. The four remaining studies focused on hospitalized patients as inpatient care is critical in the chronic phase of the disease [7,18,35,36]. According to Echeverría et al [11], 30% of the patients with CD will develop CCM that is associated with high rates of inpatient care. Given the relevance of these expenses in the management of Chagas disease, the majority of the studies estimate costs for the cardiac form [18,31–35,37,38,40,41]. The economic burden of heart failure is significant. A comprehensive assessment was carried out by Cook et al [44]. estimating the annual global economic burden of heart failure across 197 countries, encompassing approximately 99% of the global population. The primary findings indicated that in 2012, expenditures related to heart failure amounted to $108 billion. Among high-income countries, heart failure expenditures constituted an average of 2.37% of total healthcare expenditures, while for medium and low-income countries, the corresponding figure was 1.26%.

The absence of direct investigation of the costs incurred by families is a noticeable characteristic of the selected studies. Even studies that present an approach from the societal perspective tend to focus only on the costs associated with lost productivity due to absenteeism or premature death [31,33,40,41]. Only one study directly investigates the out-of-pocket expenses families incur in treating Chagas disease [39]. The authors conducted a household survey for CD patients in Colombia and found that family expenditures correspond to 20% of the total direct costs. The most important components financed by the families were transport, laboratory tests, food and accommodation. This study indicates that families bear a significant portion of the financial burden of Chagas disease underscoring the need for further research on this topic [39]. The lack of studies from the household perspective contrasts with the findings for the SR conducted for malaria [26]. According to Andrade et al [26], 96% of the selected studies considered household expenditures to estimate the costs of malaria.

The primary constraint of this SR is the limited number of studies with the explicit aim of estimating the costs of Chagas disease. There is a clear gap in economic burden estimations of CD. Five of the 15 studies in our analysis were designed to provide cost estimates for economic evaluations. While these studies are essential, they typically do not encompass all relevant costs of treating Chagas disease. In addition, six studies only estimate hospital costs, and one comprises only indirect costs. Therefore, the number of studies comprehensively assessing the costs of Chagas diseases is still scarce in the literature.

A second limitation of this SR is the lack of comparability among the studies, which prevented us from synthesizing cost estimates and their composition. The selected studies were heterogeneous in terms of estimation methods, cost components, phases and forms of the diseases, and metrics of measures. Understanding the economic burden of Chagas disease and its composition is crucial for developing health policies that target more efficient care management. As the course of Chagas disease is complex, with different phases and forms, it is not easy to estimate and standardize cost components. Each phase and form of the disease requires specific treatment and procedures, which are associated with different cost levels. This lack of comparability among studies is an issue for the generalization of the results for different countries.

Finally, the scope of this SR did not include studies related to the surveillance of Chagas disease. In general, these studies refer to the cost-effectiveness evaluations of programs to control and eradicate the disease [45–49]. In addition, these expenses strongly depend on the organization of the health system, and their accountability is not disaggregated by disease.

Although this SR does not present a systematization of the costs and economic burden, it uncovers the impact of direct (medical and non-medical) and indirect costs in various societies. This occurs in both endemic and non-endemic countries owing to the importance of congenital transmission. The results of economic burden estimation are input for conducting economic evaluations of policy interventions for Chagas disease. For instance, these studies could subsidize international agencies to fund vaccine development research and more effective vector control programs. Currently, research on vaccines for Chagas Disease prevention is still premature, but specific antiparasitic therapies are promising for controlling disease progression [50]. More invasive treatments for cardiomyopathies are available, but are usually not affordable for low and middle-income countries because of their high costs [51].

## 5 Conclusion

This systematic review represents a significant advancement in defining a framework for estimating the economic burden of Chagas disease (CD) as it allowed for identifying the most frequent and expensive procedures. For CD, medical direct costs are the most critical component evaluated due to the complexity of managing the disease, especially during the chronic phase. Standardizing a minimum set of cost components for each form and phase measured by a single metric is essential to ensure comparability in both the magnitude of cost components and cost composition analysis. Given the continued neglect of CD, estimating its economic burden is an important tool for changing this context and providing support for public policies aimed at eliminating CD.

## Supporting information

S1 Table Preferred Reporting Items for Systematic reviews and Meta-Analyses (PRISMA) checklist. (DOCX)

S2 Table Search strategies. (DOCX)

S3 Table Data collection form. (XLSM)

S4 Table Quality assessment tool. (DOCX)

S5 Table List of excluded studies in phase II. (DOCX)

S6 Table List of included studies. (DOCX)

S7 Table Quality assessment by study. (DOCX)

S8 Table Cost estimation by statistics, unity of analysis, and periodicity. (DOCX)
