# Supplementary material for: The economic burden of Chagas disease: A systematic review
Source: PLoS Negl Trop Dis. 2023 Nov 22;17(11):e0011757. doi: 10.1371/journal.pntd.0011757 (PMC10699619; doi:10.1371/journal.pntd.0011757)
Supplement: S2 Table — (DOCX) [file pntd.0011757.s002.docx]

# Appendix S2. Search strategies

| Database | Search strategy | # ref. |
| --- | --- | --- |
| Pubmed | ("Costs and Cost Analysis"[MeSH Terms] OR "Economics"[MeSH Terms] OR "Cost of Illness"[MeSH Terms] OR "Cost Sharing"[MeSH Terms] OR "Cost Savings"[MeSH Terms] OR "Cost Control"[MeSH Terms] OR "Cost-Benefit Analysis"[MeSH Terms] OR "Cost Allocation"[MeSH Terms] OR "Health Care Costs"[MeSH Terms] OR "Health Expenditures"[MeSH Terms] OR "Direct Service Costs"[MeSH Terms] OR "Hospital Costs"[MeSH Terms] OR "Drug Costs"[MeSH Terms] OR disease cost*[tiab] OR cost-analysis[tiab] OR affordab*[tiab] OR economic burden*[tiab]) AND ("Chagas Disease"[MeSH Terms] OR chagas*[ti] OR chagas*[tiab] OR American Trypanosomiasis[tiab] OR South American Trypanosomiasis[tiab] OR Trypanosoma cruzi Infection*[tiab] OR (Trypanosoma cruzi[tiab] AND Infection*[tiab]) OR (American[tiab] AND Trypanosom*[tiab])) | 184 |
| Lilacs | (tw:((mh:(Chagas Disease)) OR (tw:(Chagas Disease)) OR (tw:(Enfermedad de Chagas)) OR (tw:(Doença de Chagas)) OR (tw:(Chagas' Disease)) OR (tw:(American Trypanosomiasis)) OR (tw:(South American Trypanosomiasis)) OR (tw:(Trypanosoma cruzi Infection)) OR (tw:(Trypanosoma cruzi Infections)) OR (tw:(Infección por Trypanosoma cruzi)) OR (tw:(Tripanosomiasis Americana)) OR (tw:(Tripanosomiasis Sudamericana)) OR (tw:(Infecção por Trypanosoma cruzi)) OR (tw:(Tripanossomose Sul-Americana)) OR (tw:(Tripanossomíase Americana)) OR (tw:(Tripanossomíase Sul-Americana)) OR (tw:(Mal de Chagas)))) AND (tw:((tw:(Economia Médica)) OR (mh:(Economics, Medical)) OR (tw:(Economía Médica)) OR (tw:(Medical Economics)) OR (tw:(Economia da Saúde)) OR (mh:(Health Economics)) OR (tw:(Economía de la Salud)) OR (mh:(Economics)) OR (mh:(Costs and Cost Analysis)) OR (tw:(Costos y Análisis de Costo )) OR (tw:(Custos e Análise de Custo)) OR (tw:(Cost)) OR (tw:(cost anal*)) OR (mh:(Cost of Illness)) OR (tw:(Costo de Enfermedad)) OR (tw:(Efeitos Psicossociais da Doença)) OR (tw:(Burden of Illness)) OR (tw:(Cost of Disease)) OR (tw:(Costs and Cost Analysis)) OR (tw:(Economics, Medical)) OR (tw:(Health Economics)) OR (tw:(Economics)) OR (tw:(Cost of Illness)) OR (mh:(Cost Savings)) OR (tw:(Custo Compartilhado de Seguro)) OR (tw:(Seguro de Costos Compartidos )) OR (tw:(Cost Savings)) OR (mh:(Cost Control)) OR (mh:(Cost Allocation)) OR (mh:(Health Care Costs)) OR (mh:(Hospital Costs)) OR (mh:(Health Expenditures)) OR (tw:(affordab*)))) | 116 |
| Embase | ('Chagas disease'/exp OR 'chagas disease':ti,ab OR 'chagas':ti,ab OR 'chagas infection':ti,ab OR 'chagas mazza disease':ti,ab OR 'disease, chagas':ti,ab) AND ('cost'/exp OR 'cost':ti,ab OR 'cost allocation':ti,ab OR 'cost sharing':ti,ab OR 'costs and cost analysis':ti,ab OR 'deductibles and coinsurance':ti,ab OR 'medical savings accounts':ti,ab OR 'cost analysis':ti,ab OR 'cost control'/exp OR 'audit, cost':ti,ab OR 'cost audit':ti,ab OR 'cost containment':ti,ab OR 'cost control':ti,ab OR 'cost savings':ti,ab OR 'cost of illness'/exp OR 'cost of illness':ti,ab OR 'cost of illness analysis':ti,ab OR 'economic aspects of illness':ti,ab) | 587 |
| Total |  | 887 |
| Total without duplicates |  | 774 |
