# Supplementary material for: The economic burden of Chagas disease: A systematic review
Source: PLoS Negl Trop Dis. 2023 Nov 22;17(11):e0011757. doi: 10.1371/journal.pntd.0011757 (PMC10699619; doi:10.1371/journal.pntd.0011757)
Supplement: S4 Table — (DOCX) [file pntd.0011757.s004.docx]

# Appendix S4. Quality assessment tool.

| **n** | **Questions** |
| --- | --- |
|  | ***Analytical framework*** |
| 1 | Was the question/objective of the study properly, clear and answerable? |
| 2 | Was the target population of the study clearly described? (e.g. gender, age group, spatial-regional distribution, or socioeconomic status) |
| 3 | Was the study perspective informed? (Under which perception costs will be measured – who is disbursing the monetary resources so that benefits are generated) |
| 4 | Is the chosen time horizon appropriate to include relevant costs and consequences? (If the study reports annual cost write "NA") |
|  | ***Methodology and data*** |
| 5 | Was the method adopted for calculating the costs described? |
| 6 | Do the cost components that were included in the analysis agree with the perspective adopted in the study? (Not all table components need to be included to consider item fulfillment) |
| 7 | Have the cost components been clearly described? (They must be described and presented in a disaggregated way to allow transparency and reproducibility, informing the unit of analysis) |
| 8 | Was there information about the currency and period in which the costs were collected? (Article reported the currency and year that the data is presented, regardless of whether it is in the original format or already converted to some international currency. In this question, will only be answers accepted: fully meet or do not meet) |
| 9 | If costs were collected in different periods, was there an adjustment for inflation? (If the study is cross-section write “NA”) |
| 10 | Does the study include a discount rate? |
| 11 | Have productivity costs been included? |
| 12 | Was there separation of the disease by phases/forms? |
|  | ***Analysis and reporting*** |
| 13 | Were the cost components presented in a disaggregated way in the results section? (E.g. disease subtypes, severity, stage; subpopulation groups, results presented with and without productivity costs) |
| 14 | Are all important variables appropriately subjected to sensitivity analysis? |
| 15 | Does the study discuss the generalizability of the results to other settings or patient/client groups? |
| 16 | Does the article indicate that there is no potential conflict of interest of study researcher(s) and funder(s)? |
