# Supplementary material for: The economic burden of Chagas disease: A systematic review
Source: PLoS Negl Trop Dis. 2023 Nov 22;17(11):e0011757. doi: 10.1371/journal.pntd.0011757 (PMC10699619; doi:10.1371/journal.pntd.0011757)
Supplement: S5 Table — (DOCX) [file pntd.0011757.s005.docx]

# Appendix S5. List of excluded studies in phase II.

| **Reference #** | **Country or region** | **Reason** |
| --- | --- | --- |
| 1 | Brazil | Full text not available |
| 2 | United States of America | Cost components reported were estimated in other published study |
| 3 | Brazil | Full text not available |
| 4 | Brazil | Interventional studies: social security |
| 5 | Colombia | Interventional studies: blood screening |
| 6 | Brazil | Conference abstract |
| 7 | Chile | Cost components reported were estimated in other published study |
| 8 | Mexico | Cost components reported were estimated in other published study |
| 9 | Mexico | Cost components reported were estimated in other published study |
| 10 | Mexico | Cost components reported were estimated in other published study |
| 11 | Argentina | Cost components reported were estimated in other published study |
| 12 | Bolivia | Cost components reported were estimated in other published study |
| 13 | Colombia | Same data from another study already included in the review |
| 14 | Colombia | Full text not available |
| 15 | Brazil | Does not present cost estimates |
| 16 | Spain | Interventional studies: blood screening |
| 17 | United States of America | Full text not available |
| 18 | Latin America | Cost components reported were estimated in other published study |
| 19 | Mexico | Cost components reported were estimated in other published study |
| 20 | Endemic countries | Cost components reported were estimated in other published study |
| 21 | Colombia | Does not present cost estimates |
| 22 | Latin America | Cost components reported were estimated in other published study |
| 23 | Latin America | Cost components reported were estimated in other published study |
| 24 | United States of America | Conference abstract |
| 25 | United States of America | Interventional studies: blood screening |
| 26 | Latin America | Interventional studies: benznidazole |
| 27 | France, Germany, Italy, Switzerland, and Spain | Cost components reported were estimated in other published study |
| 28 | Mexico | Interventional studies: blood screening |
| 29 | 33 countries with notified cases in the last 15 years | Commentary/Letter |
| 30 | Argentina, Brazil, Bolivia, Chile, Paraguay, Peru, and Uruguay | Cost components reported were estimated in other published study |
| 31 | Spain | Cost components reported were estimated in other published study |
| 32 | United States of America | Conference abstract |
| 33 | Argentina | Interventional studies: vector control |
| 34 | Brazil | Commentary/Letter |
| 35 | 19 countries of Latin America and the Caribbean | Cost components reported were estimated in other published study |
| 36 | United States of America | Cost components reported were estimated in other published study |
| 37 | United States of America | Full text not available |
| 38 | Brazil | Full text not available |
| 39 | Chile | Full text not available |

References

1. Abuhab A, Trindade E, Fujii SM, Bocchi EA, Bacal F. 426 Chagas Cardiomyopathy: The Economic Burden of an Expensive, Neglected Disease, and Cause for Heart Failure. The Journal of Heart and Lung Transplantation. 2012;31(4):S151. <https://doi.org/10.1016/j.healun.2012.01.436>
2. Agapova M, Busch MP, Custer B. Cost-effectiveness of screening the US blood supply for Trypanosoma cruzi. Transfusion. 2010;50(10):2220–32. <https://doi.org/10.1111/j.1537-2995.2010.02686.x>
3. Akhavan D. Análise de custo-efetividade do programa de controle da Doença de Chagas no Brasil: relatório final. 2000. Available from: <https://pesquisa.bvsalud.org/portal/resource/pt/biblio-927067>
4. Almeida O, Meirelles P, Laurentys LL. Aspectos previdenciarios da doenca de Chagas. Rev bras saúde ocup. 1983;70–3. Available from: <https://pesquisa.bvsalud.org/portal/resource/pt/lil-18754>
5. Alvis NJ, Díaz DP, Castillo L, Alvis NR, Bermúdez MI, Berrío OM, et al. Costs of Chagas’ disease screening test in blood donors in two Colombian blood banks. Biomedica: Revista Del Instituto Nacional De Salud. 2018;38(1):61–8. <https://doi.org/10.7705/biomedica.v38i0.3477>
6. Amaral LM, Fernandes RA, Takemoto MLS, Padula AC, Vasconcellos JF, Haas LC, Valle P. Brazilian program for research and development in neglected diseases: analyses of hospitalization patterns and costs. Value in Health. 2014;17(3);A271. <https://doi.org/10.1016/j.jval.2014.03.1580>
7. Apt B W, Heitmann G I, Jercic L MI, Jofré M L, Muñoz C. del V P, Noemí H I, et al. Guías clínicas de la enfermedad de Chagas: Parte I. Introducción y epidemiología. Revista chilena de infectología. 2008;25(3):189–93. <http://dx.doi.org/10.4067/S0716-10182008000300008>
8. Bartsch SM, Avelis CM, Asti L, Hertenstein DL, Ndeffo-Mbah M, Galvani A, et al. The economic value of identifying and treating Chagas disease patients earlier and the impact on Trypanosoma cruzi transmission. PLOS Neglected Tropical Diseases. 2018;12(11):e0006809. <https://doi.org/10.1371/journal.pntd.0006809>
9. Bartsch SM, Bottazzi ME, Asti L, Strych U, Meymandi S, Falcón-Lezama JA, et al. Economic value of a therapeutic Chagas vaccine for indeterminate and Chagasic cardiomyopathy patients. Vaccine. 2019 Jun;37(28):3704–14. <https://doi.org/10.1016/j.vaccine.2019.05.028>
10. Bartsch SM, Stokes-Cawley OJ, Buekens P, Asti L, Bottazzi ME, Strych U, et al. The potential economic value of a therapeutic Chagas disease vaccine for pregnant women to prevent congenital transmission. Vaccine. 2020 Apr;38(16):3261–70. <https://doi.org/10.1016/j.vaccine.2020.02.078>
11. Basombrío MA, Schofield CJ, Rojas CL, del Rey EC. A cost-benefit analysis of Chagas disease control in north-western Argentina. Transactions of the Royal Society of Tropical Medicine and Hygiene. 1998 Mar;92(2):137–43. <https://doi.org/10.1016/S0035-9203(98)90720-9>
12. Billot C, Torrico F, Carlier Y. Estudio de costo/beneficio de un programa de control de enfermedad de Chagas congénita en Bolivia. Revista-Sociedade Brasileira De Medicina Tropical. 2005;38:108. Available from: <https://www.researchgate.net/profile/Yves-Carlier/publication/7293930_Cost_effectiveness_study_of_a_control_program_of_congenital_Chagas_disease_in_Bolivia/links/57220fa208aee491cb32c49b/Cost-effectiveness-study-of-a-control-program-of-congenital-Chagas-disease-in-Bolivia.pdf>
13. Castillo-Riquelme M, Chalabi Z, Lord J, Guhl F, Campbell-Lendrum D, Davies C, et al. Modelling geographic variation in the cost-effectiveness of control policies for infectious vector diseases: The example of Chagas disease. Journal of Health Economics. 2008;27(2):405–26. <https://doi.org/10.1016/j.jhealeco.2007.04.005>
14. Cucunuba ZM, Sicuri E, Diaz D, Basanez MG, Nouvellet, P, Conteh, L. Estimating the costs and cost-effectiveness of early diagnosis and treatment of Chagas Disease in Colombia. American Journal of Tropical Medicine and Hygiene. 2016;95(5_Suppl), 364-364. <https://doi.org/10.4269/ajtmh.program2016>
15. França SB, Abreu DMX de. Morbidade hospitalar por doença de Chagas no Brasil. Revista da Sociedade Brasileira de Medicina Tropical. 1996 Apr;29(2):109–15. <https://doi.org/10.1590/S0037-86821996000200003>
16. Sánchez Gómez A, Aparicio P, Abad Á. Enfermedad de Chagas en España: valoración de un programa de cribado de la infección congénita. 2007;9(3):130–7. Available from: <http://enfermedadesemergentes.com/articulos/a541/s-9-3-005.pdf>
17. Jin C, Srikanth Yandrapalli, Aronow WS. Chagas heart disease in the United States: a national study from 2003-2017. 2021 May 1;77(18):849–9. Available from: <https://www.jacc.org/doi/full/10.1016/S0735-1097%2821%2902208-7>
18. Lee BY, Bacon KM, Connor DL, Willig AM, Bailey RL. The Potential Economic Value of a Trypanosoma cruzi (Chagas Disease) Vaccine in Latin America. 2010 Dec 14;4(12):e916–6. <https://doi.org/10.1371/journal.pntd.0000916>
19. Lee BY, Bacon KM, Wateska AR, Bottazzi ME, Dumonteil E, Hotez PJ. Modeling the economic value of a Chagas’ disease therapeutic vaccine. Human Vaccines & Immunotherapeutics. 2012 Sep 16;8(9):1293–301. <https://doi.org/10.4161/hv.20966>
20. Lenk EJ, Redekop WK, Luyendijk M, Fitzpatrick C, Niessen LW, Stolk WA, et al. Socioeconomic benefit to individuals of achieving 2020 targets for four neglected tropical diseases controlled/eliminated by innovative and intensified disease management: Human African trypanosomiasis, leprosy, visceral leishmaniasis, Chagas disease. 2018 Mar 13;12(3):e0006250–0. <https://doi.org/10.1371/journal.pntd.0006250>
21. Marchiol A, Forsyth C, Bernal O, Hernández CV, Cucunubá Z, Abril EP, et al. Increasing access to comprehensive care for Chagas disease: development of a patient-centered model in Colombia. Revista Panamericana de Salud Pública [Internet]. 2018;41:e153. <https://doi.org/10.26633/RPSP.2017.153>
22. Moncayo A. Chagas disease: current epidemiological trends after the interruption of vectorial and transfusional transmission in the Southern Cone countries. Memórias do Instituto Oswaldo Cruz. 2003 Jul;98(5):577–91. <https://doi.org/10.1590/S0074-02762003000500001>
23. Moncayo Á, Silveira AC. 4 - Current epidemiological trends of Chagas disease in Latin America and future challenges: epidemiology, surveillance, and health policies. ScienceDirect. London: Elsevier. 2017:59–88. <https://doi.org/10.1016/B978-0-12-801029-7.00004-6>
24. Nolan MS, Hotez P, Woc-Colburn L, Murray K. Risk factors and seroprevalence of trypanosoma cruzi infection in Texas. American Journal of Tropical Medicine and Hygiene. 2013;89(5_Suppl):442. Available from: <https://www.embase.com/records?subaction=viewrecord&rid=2&page=1&id=L71313281>
25. Perez-Zetune V, Bialek SR, Montgomery SP, Stillwaggon E. Congenital Chagas Disease in the United States: The Effect of Commercially Priced Benznidazole on Costs and Benefits of Maternal Screening. The American Journal of Tropical Medicine and Hygiene [Internet]. 2020 May 1;102(5):1086–9. <https://doi.org/10.4269/ajtmh.20-0005>
26. Pinheiro E, Brum-Soares L, Reis R, Cubides JC. Chagas disease: review of needs, neglect, and obstacles to treatment access in Latin America. Revista da Sociedade Brasileira de Medicina Tropical. 2017 Jun;50(3):296–300. <https://doi.org/10.1590/0037-8682-0433-2016>
27. Requena-Méndez A, Bussion S, Aldasoro E, Jackson Y, Angheben A, Moore D, et al. Cost-effectiveness of Chagas disease screening in Latin American migrants at primary health-care centres in Europe: a Markov model analysis. The Lancet Global Health. 2017;5(4), e439-e447. <https://doi.org/10.1016/S2214-109X(17)3007>
28. Sánchez-González G, Figueroa-Lara A, Elizondo-Cano M, Wilson L, Novelo-Garza B, Valiente-Banuet L, et al. Cost-Effectiveness of Blood Donation Screening for Trypanosoma cruzi in Mexico. Carvalho MS, editor. PLOS Neglected Tropical Diseases. 2016 Mar 22;10(3):e0004528. <https://doi.org/10.1371/journal.pntd.0004528>
29. Schmuñis G. Status of and cost of Chagas disease worldwide. The Lancet infectious diseases. 2013;13(4), 283-284. <https://doi.org/10.1016/S1473-3099(13)70032-X>
30. Schofield CJ, Dias JCP. A cost-benefit analisys of chagas disease control. Memórias do Instituto Oswaldo Cruz. 1991 Sep;86(3):285–95. <https://doi.org/10.1590/S0074-02761991000300002>
31. Sicuri E, Muñoz J, Pinazo MJ, Posada E, Sanchez J, Alonso PL, et al. Economic evaluation of Chagas disease screening of pregnant Latin American women and of their infants in a non endemic area. Acta Tropica. 2011 May;118(2):110–7. <https://doi.org/10.1016/j.actatropica.2011.02.012>
32. Stillwaggon E, Perez-Zetune V. Minimizing the cost of congenital Chagas disease in the United States through maternal screening. American Journal of Tropical Medicine and Hygiene. 2017;97(5_Suppl). <https://doi.org/10.4269/ajtmh.program2017>
33. Vazquez-Prokopec GM, Spillmann C, Zaidenberg M, Kitron U, Gürtler RE. Cost-Effectiveness of Chagas Disease Vector Control Strategies in Northwestern Argentina. Lehane MJ, editor. PLoS Neglected Tropical Diseases. 2009 Jan 20;3(1):e363. <https://doi.org/10.1371/journal.pntd.0000363>
34. Veloso HH. Cost-effectiveness analysis of in-hospital heart failure treatment of Chagas' cardiomyopathy in comparison to other etiologies. International journal of cardiology. 2014;174(3), 872. <https://doi.org/10.1016/j.ijcard.2014.04.213>
35. Wilson LS, Strosberg AM, Barrio K. Cost-effectiveness of Chagas disease interventions in Latin America and the Caribbean: Markov models. The American Journal of Tropical Medicine and Hygiene. 2005 Nov 1;73(5):901–10. Available from: <https://citeseerx.ist.psu.edu/document?repid=rep1&type=pdf&doi=ea2eb5ec1781864e3016006177eb0b8385d78a4a>
36. Wilson LS, Ramsey JM, Koplowicz YB, Valiente-Banuet L, Motter C, Bertozzi SM, Tobler LH. Cost-effectiveness of implementation methods for ELISA serology testing of Trypanosoma cruzi in California blood banks. The American journal of tropical medicine and hygiene. 2008;79(1), 53-68. Available from: <https://www.researchgate.net/profile/Janine-Ramsey/publication/5245759_Cost-effectiveness_of_Implementation_Methods_for_ELISA_Serology_Testing_of_Trypanosoma_cruzi_in_California_Blood_Banks/links/5516bbff0cf2b5d6a0f00a61/Cost-effectiveness-of-Implementation-Methods-for-ELISA-Serology-Testing-of-Trypanosoma-cruzi-in-California-Blood-Banks.pdf>
37. Yandrapalli S, Tariq S, Harikrishnan P, Vuddanda VLK, Sanaani A, Aronow W, et al. Chagas Heart Disease: A United States National Study. Journal of the American College of Cardiology. 2017;11(69), 941. Available from: <https://www.researchgate.net/publication/315318283_CHAGAS_HEART_DISEASE_A_UNITED_STATES_NATIONAL_STUDY>
38. Zicker F, Zicker EMS. Benefícios previdenciários por incapacidade como indicador de morbilidade: estudo da doença de Chagas em Goiás. Rev goiana med. 1985;125–36. Available from: <https://pesquisa.bvsalud.org/portal/resource/pt/lil-42119>
39. Zurita R. JC, Andrade L. R, Tapia Ñ. C, Zavala V. F, Lorca H. M. Distribución y forma de presentación clínica del megacolon chagßsico en 96 pacientes serológicamente positivos a Trypanosoma Cruzi del Hospital San Juan de Dios: Santiago, Chile: 1990-2000. Bol Hosp San Juan de Dios. 2003;347–50. Available from: <https://pesquisa.bvsalud.org/portal/resource/pt/lil-390476>
