# Supplementary material for: The economic burden of Chagas disease: A systematic review
Source: PLoS Negl Trop Dis. 2023 Nov 22;17(11):e0011757. doi: 10.1371/journal.pntd.0011757 (PMC10699619; doi:10.1371/journal.pntd.0011757)
Supplement: S6 Table — (DOCX) [file pntd.0011757.s006.docx]

# Appendix S6. List of included studies.

| **Ref #** | **Study** | **Year of study** | **Country** | **Population** | **Currency/Year** | **Disease state/form** | **Design** | **Source of cost data** | **Cost components estimated** | **Funder** |
| --- | --- | --- | --- | --- | --- | --- | --- | --- | --- | --- |
| 7 | Herrador et al., 2015 | 1997-2011 | Spain | Mothers / General population | Euro/2011 |  | Non-Model-based | Estimated by the authors | Hospitalization (Total) |  |
| 17 | Oliveira et al., 2021 | 2010-2017 | Colombia | Adults | USD/2017 | Not specified | Non-Model-based | Estimated by the authors | Mortality ($) |  |
| 18 | Castillo-Riquelme et al., 2008 | 2004 | Colombia | General population | USD/2004 | Cardiac | Non-Model-based | Estimated by the authors / Medical records | Ambulatory/Outpatient care / Diagnosis / Drugs/Medicines (Not specified) / Electrophysiological study / Eletrocardiogram (ECG) / Emergency / Exams (Total Laboratorial tests) / Hospitalization (ICU) / Hospitalization (Not specified) / Hospitalization (Total) / Other Medical direct cost / Surgery (Not specified) / Total (Medical direct cost) | Wellcome Trust |
| 19 | Hasslocher-Moreno et al., 2013 | 2009-2011 | Brazil | General population | Real/2012 |  | ABC and DEA | Administrative records of payments | Ambulatory/Outpatient care / Consultation (Specialist - other) / Exams (Total Image) / Exams (Total Laboratorial tests) / Hospitalization (Bed) / Hospitalization (ICU) / Hospitalization (Not specified) / Other Medical direct cost / Other Non-medical direct cost |  |
| 31 | Lee et al., 2013 | Not specified | 33 countries with notified cases in the last 15 years | General population | USD/2012 | Acute, Indeterminate, Cardiac, and Digestive | Markov model | Estimated by the authors / Other articles | Total (Medical direct cost) / Surgery (Megaconlon) / Pacemaker (Not specified) / Total (Medical direct cost) / Other Indirect costs / Total cost (Medical direct and indirect) | Bill & Melinda Gates Foundation; National Institute of General Medical Sciences Models of Infectious Disease Agent Study |
| 32 | Schenone, 1998 | 1998 | Chile | General population | USD/1998 | Cardiac | Non-Model-based | Administrative records of payments | Pacemaker (Prosthesis) / Chest X-ray / Eletrocardiogram (ECG) / Enema opaco/Barium enema / Serological test / Other Medical direct cost / Total (Medical direct cost) / Exams (Total Laboratorial tests) / Enalapril / Digoxina / Furosemida / Hospitalization (Total) / Hospitalization (Bed) / Pacemaker (Implantation/Surgery) / Material/Inputs / Drugs/Medicines (Not specified) / Other / Exams (Total) / Pacemaker (Prosthesis + Implantation/Surgery) / Amiodarone / Surgery (Achalasia) / Colostomy / Echocardiogram/Cardiac ultrasonography / Holter / Ergometric Test |  |
| 33 | Abad-Franch & Aguilar, 2003 | Not specified | Ecuador | General population | USD/2003 | Acute, Indeterminate, Cardiac, and Digestive | Non-Model-based | Estimated by the authors/ Other articles | Total cost (Medical direct and indirect)/ Mortality ($) | Ministry of Public Health of Ecuador |
| 34 | Vallejo et al., 2002 | Not specified | Mexico | General population | USD/1998 | Cardiac | Non-Model-based | Estimated by the authors / Medical records | Hospitalization (Total) / Total (Medical direct cost) / Pacemaker (Prosthesis + Implantation/Surgery) |  |
| 35 | Abuhab et al., 2013 | 2006-2011 | Brazil | Adults | USD/2006 | Cardiac | Non-Model-based | Medical records | Hospitalization (Total)/ Hospitalization (Not specified) / Drugs/Medicines (Not specified) / Exams (Total) / Surgery (Not specified) |  |
| 36 | Aguirre-Salegui & Sarría-Urigüen, 2018 | 2010-2015 | Spain | General population | Euro/2015 | Not specified | Non-Model-based | Administrative records of payments | Hospitalization (Total) |  |
| 37 | Castillo-Riquelme et al., 2013 | 2012 | Chile | Mothers / Newborns/children / Not specified | Chilean peso/2012 | Acute, Indeterminate, Cardiac, Digestive, and Mixed | Markov model | Estimated by the authors | Total (Medical direct cost) / Amiodarone / Chest X-ray / Colon resection / Consultation (Other) / Consultation (Other) / Consultation (Specialist - not specified) / Consultation (Specialist - other) / Diagnosis / Echocardiogram/Cardiac ultrasonography / Eletrocardiogram (ECG) / Enema opaco/Barium enema / Ergometric Test / Exams (Total Image) / Holter / Hospitalization (Bed) / Hospitalization (ICU) / Hospitalization (Not specified) / Nifurtimox (Etiological treatment) / Other Medical direct cost / Pacemaker (Implantation/Surgery) / Pacemaker (Prosthesis + Implantation/Surgery) / Screening / Surgery (Achalasia) / Total (Medical direct cost) | Ministry of Health of Chile |
| 38 | Imaz-Igleia et al., 2015 | 2013 | Spain | Adults / Baby/Newborns / Children / Mothers / Not specified / Other | Euro/2013 | Acute, Indeterminate, Cardiac, and Digestive | Decision tree | Estimated by the authors / Official data | Adverse events / Benznidazole/Rochagan (Etiological treatment) / Diagnosis / Nifurtimox (Etiological treatment) / Other Non-medical direct cost / Total (Non-medical direct cost) / Cardiac ultrasonography / Chest X-ray / Consultation (Cardiologist) / Consultation (Gastroenterologist) / Consultation (Not specified) / Defibrillator / Digoxina / Drugs/Medicines (Not specified) / ECG + STRESS TEST / Eletrocardiogram (ECG) / Enalapril / Exams (Total Image) / Furosemida / Holter / Hospitalization (Not specified) / Hospitalization (Total) / Opportunity costs (Medical care) / Opportunity costs (Patient absenteeism) / Other Non-medical direct cost / Pacemaker (Prosthesis + Implantation/Surgery) / Pacemaker (Prosthesis) / Surgery (Megaviscera) / Total (Medical direct cost) / Total cost (Non-medical direct and indirect) / Transplant / Traveling costs (Bus fare) |  |
| 39 | Oliveira & Buitrago, 2020 | 2017 | Colombia | Adults | USD/2017 | Not specified | Non-Model-based | Official data / Patient interviews / Other | Total (Medical direct cost) / Other Medical direct cost / Other Non-medical direct cost / Opportunity costs (Patient presenteeism) / Mortality ($) / Total cost (Medical/non-medical direct and indirect) / Exams (Total Laboratorial tests) / Total (Non-medical direct cost) / Consultation (Home visits) / Hospitalization (Total) / Traveling costs (Bus fare) / Diagnosis / Consultation (General practioner) / Emergency / Opportunity costs (Patient absenteeism) / Consultation (Specialist - not specified) / Lodging / Total (Indirect cost) / Food | National Institute of Health of Colombia; Faculty of Medicine of the National University of Colombia |
| 40 | Ramsey et al., 2014 | Not specified | Mexico | General population | USD/2012 | Acute, Indeterminate, Cardiac, and Digestive | Markov model | Experts / Estimated by the authors / Other articles | Exams (Total Image) / Hospitalization (Not specified) / Opportunity costs (Patient absenteeism) / Drugs/Medicines (Not specified) / Consultation (Not specified) / Pacemaker (Implantation/Surgery) / Screening / Surgery (Megaconlon) / Exams (Total Laboratorial tests) / Total cost (Medical direct and indirect) / Consultation (Specialist - not specified) | CONACyT-FONSEC |
| 41 | Stillwaggon et al., 2018 | Not specified | United States of America | Mothers / Baby/Newborns / Not specified | USD/2016 | Indeterminate, Cardiac, and Digestive | Decision tree | Estimated by the authors / Official data | Chest X-ray / ECG + STRESS TEST / Total cost (Medical direct and indirect) / Fundoplication / Consultation (Gastroenterologist) / Pacemaker (Implantation/Surgery) / Transplant / Other / Consultation (Cardiologist) / Screening / Amiodarone / Esophageal relaxants / Mortality ($) / Hospitalization (Not specified) / Colon resection / Laxatives / Consultation (General practioner) |  |
