# Supplementary material for: The economic burden of Chagas disease: A systematic review
Source: PLoS Negl Trop Dis. 2023 Nov 22;17(11):e0011757. doi: 10.1371/journal.pntd.0011757 (PMC10699619; doi:10.1371/journal.pntd.0011757)
Supplement: S7 Table — (DOCX) [file pntd.0011757.s007.docx]

# Appendix S7. Quality assessment by study.

| **Items Assessed** | **Study** | | | | | | | | | | | | | | |
| --- | --- | --- | --- | --- | --- | --- | --- | --- | --- | --- | --- | --- | --- | --- | --- |
|  | **7** | **17** | **18** | **19** | **31** | **32** | **33** | **34** | **35** | **36** | **37** | **38** | **39** | **40** | **41** |
| Objective clearly stated and properly answered | Yes | Yes | Yes | Yes | Yes | Partially | Yes | Yes | Yes | Yes | Yes | Yes | Yes | Yes | Yes |
| Target population clearly described | Yes | Yes | Yes | Yes | Yes | Partially | Partially | Yes | Yes | Yes | Yes | Yes | Yes | Yes | Yes |
| Study perspective stated | Yes | Yes | Yes | Yes | Yes | Yes | No | Yes | Yes | Yes | Yes | Yes | Yes | Yes | Yes |
| Time horizon appropriate | No | NA | NA | NA | NA | NA | Unclear | Yes | Yes | Yes | Yes | Yes | NA | Yes | Yes |
| Method for costs estimation clearly described | Partially | Yes | Yes | Yes | Yes | Yes | Partially | Yes | Yes | Yes | Yes | Yes | Yes | Yes | Yes |
| Cost components in line with the study perspective | Yes | Yes | Yes | Yes | Yes | Partially | Yes | Yes | Yes | Yes | Yes | Yes | Yes | Yes | Yes |
| Cost components clearly described | Partially | Partially | Yes | Yes | Yes | Partially | Partially | Partially | Partially | No | Yes | No | Partially | Yes | Yes |
| Information on the currency and the period in which the costs were collected | No | Yes | Yes | Yes | Yes | Yes | No | Yes | Yes | Yes | Yes | Yes | Yes | Yes | Yes |
| Adjustment for inflation | No | No | NA | NA | No | NA | Unclear | NA | No | No | Yes | Yes | NA | Yes | NA |
| Discount rate | No | Yes | Yes | NA | Yes | NA | No | No | No | No | Yes | Yes | Yes | Yes | Yes |
| Productivity costs stated | No | Yes | No | No | Yes | No | Yes | No | No | No | No | Yes | Yes | Yes | Yes |
| Separation in disease phases/forms | No | No | Partially | No | Yes | Partially | Yes | Partially | Partially | No | Yes | Yes | No | Yes | Yes |
| Cost components results presented in a disaggregated way | No | NA | Yes | Yes | Yes | No | Partially | Partially | Partially | No | NA | Yes | Partially | Yes | NA |
| Sensitivity analysis performed | No | Yes | No | No | Yes | No | No | No | No | No | Yes | Yes | Yes | No | Yes |
| Generalization of the results discussed | No | No | Yes | No | Yes | No | No | No | Yes | No | No | No | Partially | No | Yes |
| Conflict of interest stated | Yes | Yes | Yes | No | Yes | No | No | No | No | Yes | Yes | No | Yes | Yes | Yes |
