# Supplementary material for: The economic burden of Chagas disease: A systematic review
Source: PLoS Negl Trop Dis. 2023 Nov 22;17(11):e0011757. doi: 10.1371/journal.pntd.0011757 (PMC10699619; doi:10.1371/journal.pntd.0011757)
Supplement: S8 Table — (DOCX) [file pntd.0011757.s008.docx]

# Appendix S8. Cost estimation by statistics, unity of analysis, and periodicity.

| **Metric** | **# studies** | **Reference** |
| --- | --- | --- |
| **Mean per patient** |  |  |
| annual | 8 | [18,19,31,32,33,34,39,40] |
| lifetime | 4 | [18,31,38,40] |
| not specified/not aplicable | 1 | [33] |
| per period | 1 | [38] |
| **Mean per unity** |  |  |
| annual | 2 | [19,33] |
| **Mean total** |  |  |
| annual | 3 | [19,31,33] |
| lifetime | 1 | [31] |
| **Min per patient** |  |  |
| annual | 3 | [31,32,34] |
| lifetime | 2 | [31,38] |
| not specified/not aplicable | 2 | [32,34] |
| monthly | 1 | [32] |
| **Min total** |  |  |
| annual | 3 | [31,32,34] |
| lifetime | 1 | [31] |
| **Max per patient** |  |  |
| annual | 4 | [18,31,32,34] |
| lifetime | 2 | [31,38] |
| not specified/not aplicable | 1 | [34] |
| **Max total** |  |  |
| annual | 3 | [31,32,34] |
| lifetime | 1 | [31] |
| **Median per patient** |  |  |
| not specified/not aplicable | 1 | [35] |
| per day | 1 | [35] |
| **Median per unity** |  |  |
| not specified/not aplicable | 1 | [7] |
| **Total per patient** |  |  |
| annual | 3 | [33,37,39] |
| lifetime | 2 | [33,41] |
| not specified/not aplicable | 1 | [37] |
| per period | 1 | [17] |
| **Total total** |  |  |
| annual | 5 | [17,18,33,37,39] |
| lifetime | 1 | [33] |
| per period | 1 | [17] |
| **Other** | 2 | [17,40] |
